# High-fat meals rich in EPA plus DHA compared with DHA only have differential effects on postprandial lipemia and plasma 8-isoprostane F_2α_ concentrations relative to a control high–oleic acid meal: a randomized controlled trial^1^,^2^,^3^,^4^

**DOI:** 10.3945/ajcn.114.091223

**Published:** 2014-08-06

**Authors:** Robert Purcell, Sally H Latham, Kathleen M Botham, Wendy L Hall, Caroline PD Wheeler-Jones

**Affiliations:** 1 From Comparative Biomedical Sciences, Royal Veterinary College (RP, SHL, KMB, and CPDW-J) and Diabetes & Nutritional Sciences Division (WLH), King's College London, London, United Kingdom.

## Abstract

**Background:** Eicosapentaenoic acid (EPA) plus docosahexaenoic acid (DHA) supplementation has beneficial cardiovascular effects, but postprandial influences of these individual fatty acids are unclear.

**Objectives:** The primary objective was to determine the vascular effects of EPA + DHA compared with DHA only during postprandial lipemia relative to control high–oleic acid meals; the secondary objective was to characterize the effects of linoleic acid–enriched high-fat meals relative to the control meal.

**Design:** We conducted a randomized, controlled, double-blind crossover trial of 4 high-fat (75-g) meals containing *1*) high–oleic acid sunflower oil (HOS; control), *2*) HOS + fish oil (FO; 5 g EPA and DHA), *3*) HOS + algal oil (AO; 5 g DHA), and *4*) high–linoleic acid sunflower oil (HLS) in 16 healthy men (aged 35–70 y) with higher than optimal fasting triacylglycerol concentrations (mean ± SD triacylglycerol, 1.9 ± 0.5 mmol/L).

**Results:** Elevations in triacylglycerol concentration relative to baseline were slightly reduced after FO and HLS compared with the HOS control (*P* < 0.05). The characteristic decrease from baseline in plasma nonesterified fatty acids after a mixed meal was inhibited after AO (Δ 0–3 h, *P* < 0.05). HLS increased the augmentation index compared with the other test meals (*P* < 0.05), although the digital volume pulse–reflection index was not significantly different. Plasma 8-isoprostane F_2α_ analysis revealed opposing effects of FO (increased) and AO (reduced) compared with the control (*P* < 0.05). No differences in nitric oxide metabolites were observed.

**Conclusions:** These data show differential postprandial 8-isoprostane F_2α_ responses to high-fat meals containing EPA + DHA–rich fish oil compared with DHA-rich AO, but these differences were not associated with consistent effects on postprandial vascular function or lipemia. More detailed analyses of polyunsaturated fatty acid–derived lipid mediators are required to determine possible divergent functional effects of single meals rich in either DHA or EPA. This trial was registered at clinicaltrials.gov as NCT01618071.

## INTRODUCTION

Long-chain (LC)^5^ n−3 PUFAs—namely, EPA (20:5n−3) and DHA (22:6n−3)—are abundant in oily fish, the main dietary source of preformed EPA and DHA. However, alternative sources are increasingly being considered as important dietary contributors of LC n−3 PUFAs for the future, including marine microalgae, krill, and genetically modified crops (1). Prospective cohort studies indicate that LC n−3 PUFA or fish intakes are associated with a lowered risk of cardiovascular disease (CVD) mortality (2), whereas evidence from randomized controlled trials is less coherent (3–5). Heterogeneous outcomes from LC n−3 PUFA trials may be influenced by a number of factors, including background diet (6), dose, medication (eg, statins) (7), concomitant risk factors, and varying ratios of EPA to DHA in fish-oil supplements. There is little information regarding the individual effects of EPA and DHA (8), and this knowledge is urgently required now that separate algal sources of DHA and EPA are commercially available or under development.

An exaggerated postprandial triglyceride-rich lipoprotein response, including chylomicrons and their remnants, is considered a key factor in the development of atherosclerosis (9). Postprandial inflammation involving endothelial cell and leukocyte activation is likely to contribute to this (10); we, as well as others, have suggested potential dependence on chylomicron/chylomicron remnant fatty acid composition (11, 12). Postprandial impairment in endothelial function after high-fat meals has been widely reported (13, 14) and may be linked to oxidative stress (15), with implications for the atherogenic potential of habitual consumption of high-fat foods. The relative postprandial effects of different unsaturated fatty acid–rich meals (MUFA compared with n−6 PUFA) require clarification (13, 14). Evidence is also accumulating that supplementation with EPA as opposed to DHA, in chronic dietary interventions, is likely to exert differential effects on outcomes such as plasma triacylglycerol concentrations, arterial stiffness, lipoprotein particle size, blood pressure, and heart rate (16), but associated mechanisms are unclear.

The primary objective of this acute randomized, controlled, double-blind crossover trial was to determine whether there were divergent effects of EPA + DHA–rich (fish oil; FO), DHA-rich (algal oil; AO), and linoleic acid (n−6 PUFA)–rich (high–linoleic acid sunflower oil; HLS) meals on vascular function and oxidative stress compared with a control MUFA-rich (high–oleic acid sunflower oil; HOS) meal. HOS was allocated as the control because it has been shown to induce pronounced postprandial lipemia and impair endothelial function (17). It was hypothesized that high-fat meals containing FO and AO would result in improved vascular and oxidative stress responses during postprandial lipemia, whereas the response to HLS would be unchanged relative to the control (HOS) in men.

## SUBJECTS AND METHODS

This single-center dietary intervention study was conducted at King's College London, United Kingdom, between June and October 2012. Ethical approval was obtained from the National Research Ethics Service Committee London – Dulwich (REC reference: 11/LO/0116). All participants signed an informed consent form before any measurements were taken. This study was conducted in accordance with the principles outlined in the Declaration of Helsinki and has been registered with clinicaltrials.gov (NCT01618071).

### Subjects

Nonsmoking men aged 35–70 y were recruited by internal e-mail at King's College London and through advertisement in free newspapers distributed London-wide. Respondents were initially interviewed by telephone to assess eligibility and then invited to attend a screening visit in the fasting state for measurement of height, weight, waist circumference, and seated blood pressure, and a blood sample was taken for liver function tests, hematology, glucose, and lipid profile. Inclusion criteria were as follows: healthy, male, aged 35–70 y, nonsmoking, and plasma triacylglycerol concentrations >1.2 mmol/L. Exclusion criteria were a reported history of CVD, cancer, kidney, liver, or bowel disease; presence of a gastrointestinal disorder or use of drugs likely to alter gastrointestinal motility; history of substance abuse or alcoholism; current self-reported alcohol intake >224 g/wk; allergy or intolerance to any component of test meals; unwillingness to restrict consumption of oily fish/FO supplements; weight change >3 kg in previous 2 mo; BMI (in kg/m^2^) <20 or >35; fasting blood cholesterol >7.8 mmol/L; and current use of lipid-lowering medication. Participants were randomly allocated to treatment order after confirmation of eligibility by using the random-number generator function RANDBETWEEN (0,100) in Microsoft Excel software.

### Test meal composition

Each participant consumed an isoenergetic high-fat meal (4.6 MJ, 75 g fat, 92.8 g carbohydrate, and 14.4 g protein) on 4 separate study visits, containing high MUFA oil (reference meal), high n−6 PUFA oil, high MUFA oil with 5 g EPA + DHA, or high MUFA oil with 5 g DHA only (**Table 1**). The test meal consisted of a muffin (3.7 MJ, 64.5 g fat, 68 g carbohydrate, and 7.6 g protein) and a strawberry-flavored (Nesquik; Nestlé) milkshake (0.9 MJ, 10.5 g fat, 24.8 g carbohydrate, and 6.8 g protein). The reference test meal (HOS meal) contained high MUFA fat in the form of HOS (ADM Trading Ltd), which was added to the muffin (64.5 g) and the milkshake (10.5 g). The high n−6 PUFA meal (HLS meal) contained HLS (ADM Trading Ltd) added in the same quantities. The high n−3 PUFA (EPA + DHA) meal (FO meal) contained HOS (64.5 g in the muffin) and 6.7 g of a purified fish oil [Incromega TG4030-(LK); Croda Europe Ltd] to provide 5 g EPA + DHA (equivalent to that derived from approximately 400 g cooked salmon), plus 3.8 g HOS in the milkshake (10.5 g total oils). The high n−3 PUFA (DHA-only) meal (AO meal) contained HOS (64.5 g in the muffin) and 10.5 g of a marine micro–algal oil extracted from *Crypthecodinium cohnii* (DHASCO; Martek Biosciences Corporation) to provide 5 g DHA in the milkshake. The meals were labeled with a concealed code by a technician at King's College London who was not involved in the study or its analysis, and all study researchers and participants were blinded to the identity of each test meal.

**TABLE 1:** Test meal fatty acid composition*^1^*

Fatty acids	HOS	HLS	FO	AO
EPA (20:5n−3)	0	0	3.1	0
DHA (22:6n−3)	0	0	1.9	5
Docosapentaenoic acid (22:5n−3)	0	0	0.4	0.1
Oleic acid (18:1n−9)	61.7	18.8	56.2	53.1
Linoleic acid (18:2n−6)	6.2	47.2	5.6	5.3
Myristic acid (14:0)	0	0	0.2	7.3
Palmitic acid (16:0)	2.9	4.9	0.3	6.3
Palmitoleic acid (16:1)	0	0	0.6	1.4

1 All values are grams. Fatty acid compositions of the oils were measured by using gas chromatography. AO, algal oil meal; FO, fish-oil meal; HLS, high–linoleic acid sunflower oil meal; HOS, high–oleic acid sunflower oil meal.

### Study protocol

A randomized, controlled, double-blind crossover study design was used, in which participants attended the metabolic research unit at King's College London on 4 separate occasions to consume each meal, with each study day being at least 1 wk apart.

Participants were asked to avoid the consumption of oily fish and fatty acid supplementation during the 2 wk before and throughout their study period. Participants were asked to refrain from consuming high-fat foods, caffeine, alcohol, and nitrate-rich foods such as green leafy vegetables and beetroot 24 h before each study day because these dietary components may have affected postprandial lipemic response, vascular function, or nitric oxide metabolite measurements. In addition, subjects were also asked to avoid strenuous exercise 24 h before each study day, to consume a specified commercially available low-fat ready meal (<10 g fat) for their evening meal, and to fast from 2030, consuming only low-nitrate water (Buxton; Nestlé) thereafter.

Participants reported to the metabolic research unit between 0800 h and 1000 h in a fasted state. On arrival, they were weighed and then rested in a supine position for 10 min. Baseline digital volume pulse (DVP) and Arteriograph*24* (TensioMed) measurements were recorded, and then an indwelling venous cannula was fitted by a trained clinician and baseline blood samples taken for plasma triacylglycerol, cholesterol, nonesterified fatty acids (NEFAs), glucose, fatty acid, nitric oxide metabolites, and 8-isoprostane F_2α_ analysis. The test meal was consumed within 10 min, and subsequently blood was taken every hour for measurement of triacylglycerol, cholesterol, NEFAs, glucose concentrations, and total fatty acid profile analysis. Blood samples were also taken at 2, 4, and 6 h for nitric oxide metabolite and 8-isoprostane F_2α_ analysis.

### Vascular measurements

Blood pressure, arterial stiffness, and changes in the reflected pulse wave were measured by using 2 methods: *1*) an oscillometric, occlusive upper-arm cuff device called the Arteriograph*24* (TensioMed), which can be used under ambulatory conditions to indirectly calculate parameters such as aortic pulse wave velocity, augmentation index [AIx; central and peripheral (AIx_ao_ and AIx_br_, respectively)], central systolic blood pressure, and brachial systolic and diastolic blood pressure (18, 19) and *2*) DVP by photoplethysmography (PulseTrace; Micro Medical Ltd) to calculate the stiffness index (DVP-SI, m/s) and reflection index (DVP-RI, %). DVP-SI is related to large-artery stiffness and correlates closely with large-artery pulse wave velocity (20). DVP-RI is more strongly related to vascular tone of small arteries and is markedly sensitive to drugs influencing vasomotor tone (21). DVP was measured in a warm, quiet environment with the participant in a supine position after a 10-min rest. All DVP measurements were taken in triplicate and the average value calculated for each time point. DVP measurements were recorded 2, 4, and 6 h after consumption of the high-fat meal. The intraobserver coefficient of variance for within-subject measurements on 6 successive days in our laboratory was 4.9% for DVP-RI and 3.8% for DVP-SI. Participants were fitted with an Arteriograph*24* cuff to wear for the duration of each study day, with measurements being recorded every 30 min, during which subjects were asked to remain still and to avoid talking. Within-subject Arteriograph*24* measurements made 1–4 wk apart at baseline in this study (*n* = 15) correlated significantly for peripheral, central, and mean arterial blood pressure (*r* = 0.7–1.0, *P* = 0.005–<0.00001) and peripheral and central AIx (*r* = 0.4–0.9, *P* = 0.00005–0.09), demonstrating good repeatability.

### Plasma lipids, glucose, nitric oxide metabolites, and 8-isoprostane F_2α_

Blood samples for total plasma fatty acid composition, lipoprotein fraction isolation, plasma triacylglycerol, cholesterol, NEFAs, and nitrite/nitric oxide metabolite (NOx) concentrations were collected into EDTA-coated tubes and centrifuged at 1500 × *g* for 15 min at 4°C. Blood samples for glucose analysis were collected into fluoride oxalate tubes and centrifuged with the EDTA-coated tubes. Apart from plasma allocated for lipoprotein fraction isolation, plasma samples were stored at −80°C until analysis. Plasma triacylglycerol, cholesterol, NEFAs, and glucose were measured on an ILab650 automated analyzer (Instrumentation Laboratory) with commercially available enzymatic kits (Instrumentation Laboratory; WAKO NEFA-HR, WAKO Chemicals GmbH). Chylomicron and intermediate-density lipoprotein fractions were analyzed for fatty acid composition, triacylglycerol and cholesterol, and applied to cultured endothelial cells to investigate inflammatory pathways in a series of experiments to be reported in a separate study (Purcell et al, unpublished results, 2014).

The fatty acid compositions of plasma and natural oils used in the study were analyzed by gas chromatography with a one-step direct transesterification process, a method extensively described and used in the literature, allowing measurement of both lipid-bound and free fatty acids (22–24).

The most reliable measure of oxidative stress in vivo is 8-isoprostane F_2α_, which is produced by the free radical–catalyzed peroxidation of arachidonic acid (25). Here, plasma 8-isoprostane F_2α_ concentrations were measured by gas chromatography/mass spectroscopy after immunoaffinity separation, a method that is both highly sensitive and accurate (26) and has been described previously (24). Our assay is highly specific to 8-isoprostane F_2α_ and does not measure F3 or F4 isoprostanes derived from EPA or DHA. The immunoaffinity columns (Cayman Chemical) used for this analysis have very little binding to the 8-isoprostane F_2α_ isomer prostaglandin F_2α_ (>0.8%). Furthermore, prostaglandin F_2α_ and 8-isoprostane F_2α_ are separated by the gas chromatograph in our setup (data not shown); thus, any contamination with PGF_2α_ does not interfere with measurement of 8-isoprostane F_2α_ concentrations.

Plasma total NOx and nitrite were measured by ozone chemiluminescence as described previously (27). Plasma nitrate was calculated by subtracting the plasma nitrite concentration from plasma NOx.

### Statistical analysis

To detect a 6.5% difference between test meals in peripheral AIx with an 80% power and a significance level of 0.01 (2-tailed), we needed 16 subjects. This difference was based on previous studies measuring pulse wave forms within our laboratory. In addition, statistical power calculations carried out on data from a similar study in our laboratory, in middle-aged men with slightly raised fasting triacylglycerol concentrations, determined that a sample size of 16 would have 80% power to detect a difference between means of 0.67 mmol/L in triacylglycerol concentrations at a significance level of 0.01. Data were tested for normality and log-transformed where necessary. Two-factor repeated-measures ANOVA (factors were treatment and time) was used to test for significant treatment × time interactions and effects of time, as well as for significant treatment effects on change from baseline values. Where an overall treatment effect or treatment × time interaction was shown, Bonferroni post hoc tests were carried out to test which meals were different from the control meal (HOS). If data were not deemed normally distributed, they were log-transformed to normalize and then the same analysis was applied. Data analysis and graphs were produced by GraphPad Prism version 6.01 (GraphPad Software).

## RESULTS

**FIGURE 1.**
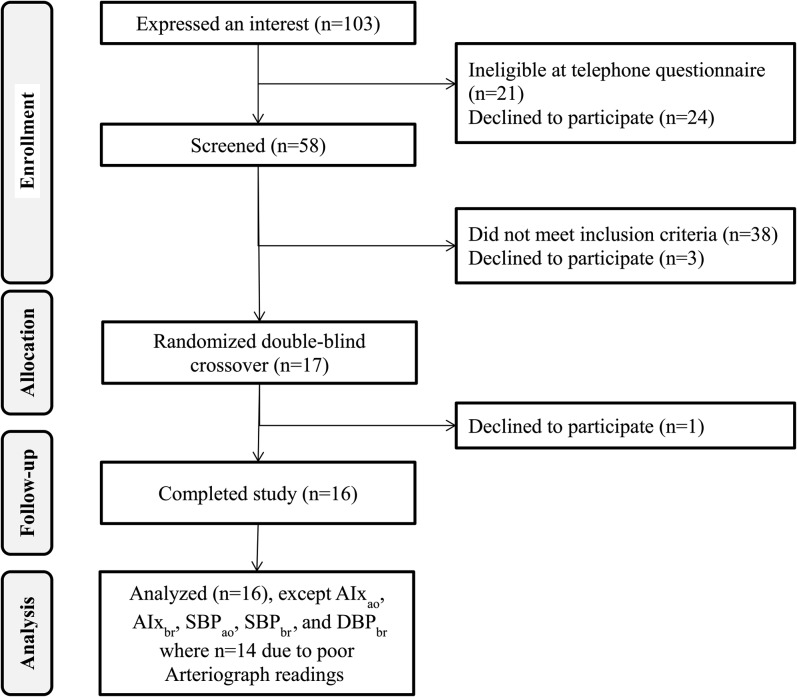
Consolidated Standards of Reporting Trials flow diagram. AIx_ao_, central augmentation index; AIx_br_, peripheral augmentation index; Arteriograph, Arteriograph *24* upper-arm cuff device (TensioMed); DBP_br_, peripheral diastolic blood pressure; SBP_ao_, central systolic blood pressure; SBP_br_, peripheral systolic blood pressure.

Sixteen subjects completed each of the 4 intervention arms (**Figure 1**). Characteristics of completing participants measured at the screening visit are shown in **Table 2**. No adverse effects were reported. Participants were mainly middle-aged (11 were aged ≥45 y), and 10 were overweight. Half met the International Diabetes Federation cutoff for abdominal adiposity (ie, waist circumference >94 cm), and 13 of 16 had fasting triacylglycerol concentrations >1.5 mmol/L.

**TABLE 2:** Participant characteristics at initial screening visit*^1^*

Characteristic	Value
Age (y)	49.9 ± 11.4
Weight (kg)	82.8 ± 14.7
Height (m)	1.74 ± 0.06
BMI (kg/m^2^)	27.3 ± 3.9
Body fat composition (%)	23.6 ± 7.6
Waist circumference (cm)	99 ± 10
Systolic blood pressure (mm Hg)	122 ± 22
Diastolic blood pressure (mm Hg)	80 ± 9
Heart rate (beats/min)	67 ± 9
Plasma triglycerides (mmol/L)	1.88 ± 0.51
Total plasma cholesterol (mmol/L)	5.26 ± 0.66
Plasma LDL cholesterol (mmol/L)	3.37 ± 0.82
Plasma HDL cholesterol (mmol/L)	1.33 ± 0.40
Plasma glucose (mmol/L)	5.46 ± 0.40

1 Values are means ± SDs. *n* = 16 participants.

### Postprandial lipids

**FIGURE 2.**
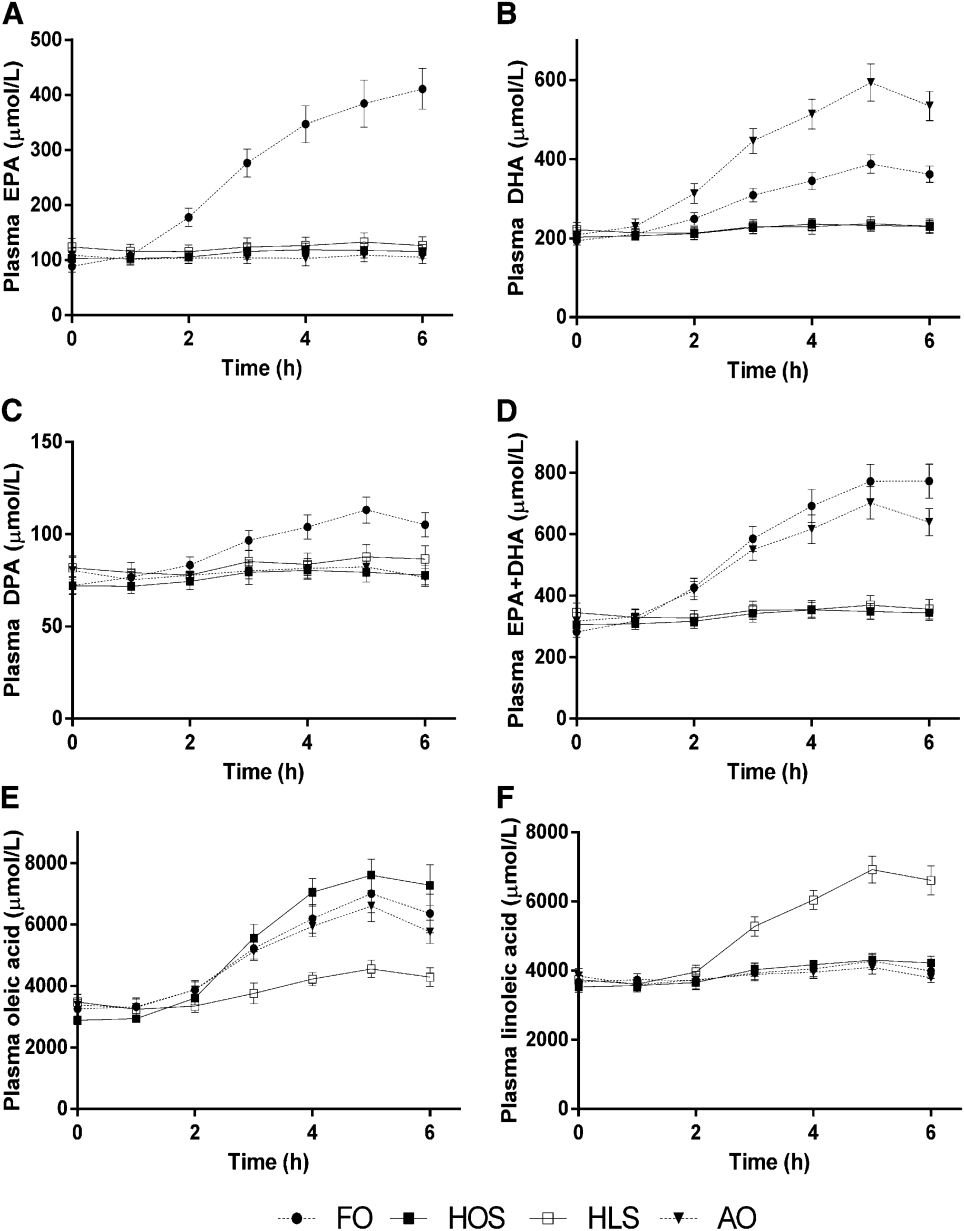
Total plasma EPA (A), DHA (B), DPA (C), EPA + DHA (D), oleic (E), and linoleic (F) fatty acids after the 4 high-fat meals. Values are expressed as means (±SEMs) as assessed by gas chromatography; *n* = 16. Each fatty acid shown changed significantly with time (*P* < 0.0001) and differed according to treatment (*P* < 0.0001), and there was a treatment × time interaction (*P* < 0.0001) as analyzed by 2-factor repeated-measures ANOVA. AO, algal oil meal; DPA, docosapentaenoic acid; FO, fish-oil meal; HLS, high–linoleic acid sunflower oil meal; HOS, high–oleic acid sunflower oil meal.

Postprandial total plasma fatty acid composition reflected meal fatty acid composition (**Figure 2**). FO resulted in a marked increase in both EPA and DHA plasma composition and also moderately increased the proportion of the fatty acids that was docosapentaenoic acid. AO induced a marked increase in DHA only, and there was a large increase in the proportion of linoleic acid after HLS (all treatment effects *P* < 0.0001). Regarding total LC n−3 PUFA (EPA + DHA), there were changes of 174% and 106% at 6 h after FO and AO, respectively.

**FIGURE 3.**
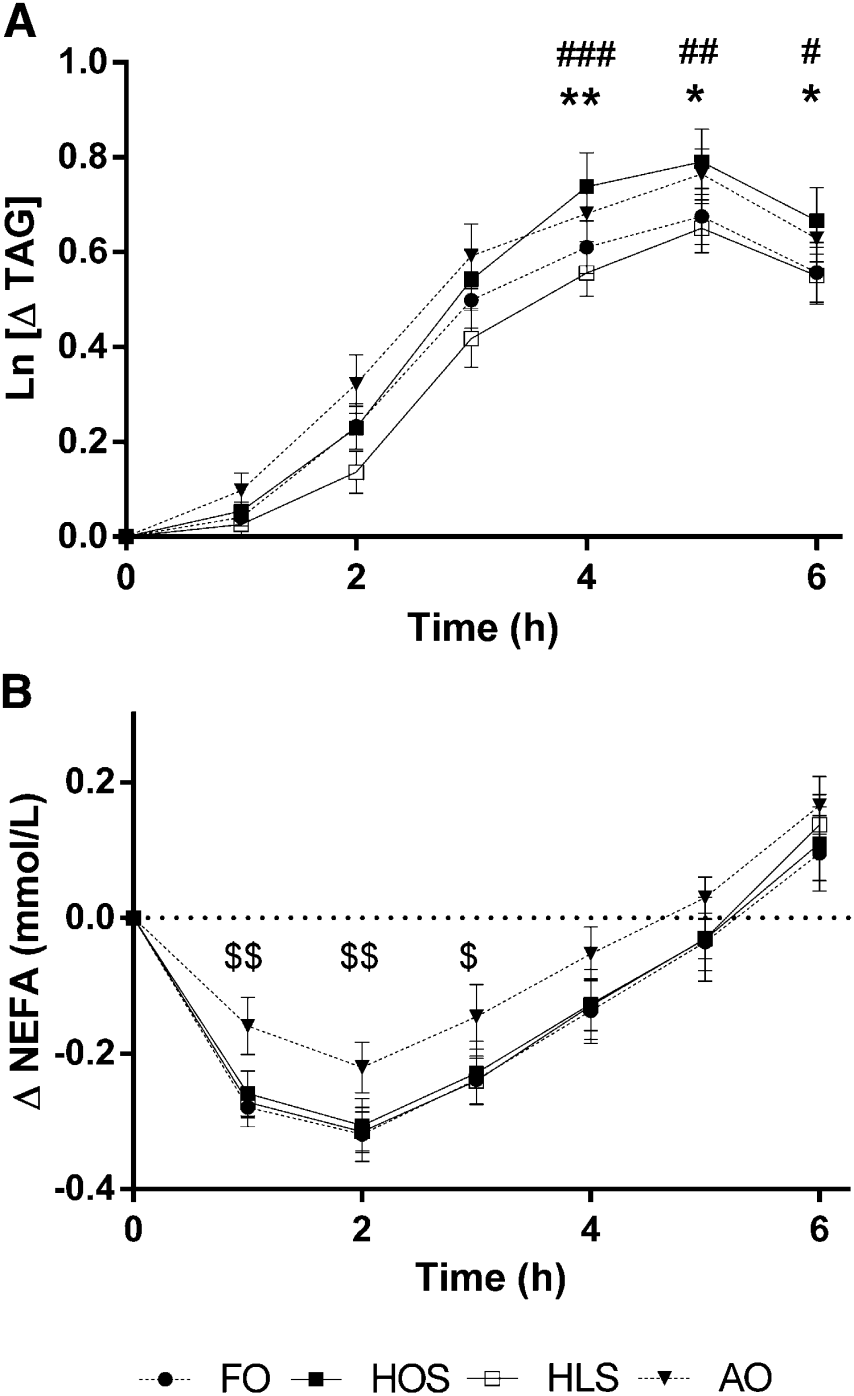
Change in postprandial plasma TAG (A) and NEFAs (B) after the 4 high-fat meals. Values for plasma TAG concentrations are mean (±SEM) changes in natural log-transformed data relative to fasting baseline values; *n* = 16. Values for plasma NEFA concentrations are mean (±SEM) changes in relation to fasting baseline values. Results were analyzed by 2-factor repeated measures ANOVA with Bonferroni post hoc analysis to determine which meals differed from control and at which time points. Both NEFAs and TAG changed with time (*P* < 0.0001) and differed according to treatment (*P* < 0.05), and there was a treatment × time interaction (*P* < 0.05). There was a reduction in (Ln)TAG after FO and HLS relative to HOS at 4, 5, and 6 h, whereas AO had no effect relative to HOS. NEFA concentrations were suppressed relative to baseline after all 4 meals, but AO diminished the reduction in NEFAs at 1, 2, and 3 h relative to HOS. FO compared with HOS: *P < 0.05, **P < 0.01; HLS compared with HOS: ^#^*P* < 0.05, ^##^*P* < 0.01, ^###^*P* < 0.001; AO compared with HOS: ^$^*P* < 0.05, ^$$^*P* < 0.01. AO, algal oil meal; FO, fish-oil meal; HLS, high–linoleic acid sunflower oil meal; HOS, high–oleic acid sunflower oil meal; NEFA, nonesterified fatty acid; TAG, triacylglycerol.

All 4 meals induced a marked increase in postprandial triacylglycerol concentrations (time effect, *P* < 0.0001), with HOS (control) inducing the greatest increase in plasma triacylglycerol concentrations, peaking at 5 h (**Figure 3A**). There was a reduction in postprandial triglyceridemia after FO and HLS relative to the control HOS meal [treatment effect on changes from baseline (0 h) to 6 h, *P* < 0.05], with post hoc comparisons showing the differences to be statistically significant at 4, 5, and 6 h, whereas AO had similar effects to HOS (post hoc statistical data shown in Figure 3 legend). Plasma NEFA concentrations decreased 1–2 h after test meals (time effect, *P* < 0.0001; Figure 3B). The magnitude of the decrease in NEFA concentrations was significantly reduced after the AO meal (treatment effect on changes from baseline 0–3 h, *P* < 0.05), with statistically significantly higher plasma NEFA concentrations evident at 1, 2, and 3 h compared with HOS. FO had no effect on plasma NEFAs relative to the HOS meal. Baseline triacylglycerol and NEFA concentrations were not different between groups (*see* Supplementary Table 1 under “Supplemental data” in the online issue). There were no differences in postprandial glucose or cholesterol concentrations between groups (data not shown).

### Vascular measurements

**FIGURE 4.**
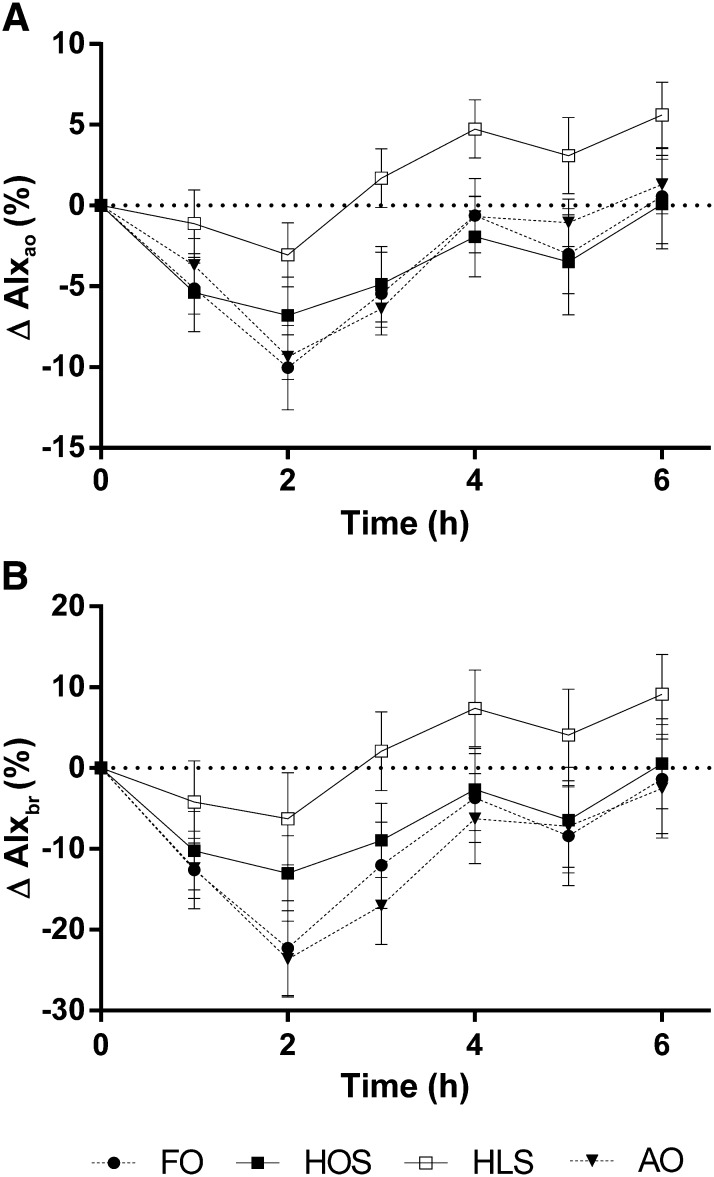
Change in aortic (A) and brachial (B) AIx after the 4 high-fat meals. Values are mean (±SEM) changes relative to fasting baseline values at 1–6 h after test meals; *n* = 14. Data were analyzed by 2-factor repeated-measures ANOVA with a Bonferroni post hoc test to determine which meals differed from control and at which time points. Both AIx_ao_ and AIx_br_ were statistically significantly different over time (*P* < 0.0001). There was a statistically significant treatment effect with AIx_ao_ (*P* < 0.05), but the effect of treatment was not significant for AIx_br_ (*P* = 0.14). There was no treatment × time interaction for either AIx_ao_ or AIx_br_. AIx_ao_, central augmentation index; AIx_br_, peripheral augmentation index; AO, algal oil meal; FO, fish-oil meal; HLS, high–linoleic acid sunflower oil meal; HOS, high–oleic acid sunflower oil meal.

There were statistically significant time effects for pulse wave reflection (AIx_ao_ and AIx_br_; *P* < 0.001), with the greatest reduction evident at the 2-h time point (**Figure 4**). Comparing the different test meals, at 2 h, the greatest decreases in pulse wave reflection were seen with FO and AO for both measures, although there was only a statistically significant overall treatment effect for AIx_ao_ (*P* < 0.05). The postprandial reduction in AIx_ao_ observed after HOS, FO, and AO was initially diminished after HLS, and after 3 h, no lowering effect was detected. There were no statistically significant differences in heart rate, central systolic blood pressure, or mean arterial pressure after the different test meals. There were no treatment differences observed in peripheral systolic or diastolic blood pressure, DVP-SI or DVP-RI, or plasma NOx (**Table 3**).

**TABLE 3:** Postprandial plasma nitric oxide metabolite concentrations and vascular measures after high-fat meals containing high–oleic acid sunflower oil plus fish oil, high–oleic acid sunflower oil plus algal oil, high–linoleic acid sunflower oil, or high–oleic acid sunflower oil only*^1^*

Meal	Baseline	*Δ*1 h	*Δ*2 h	*Δ*3 h	*Δ*4 h	*Δ*5 h	*Δ*6 h
Total NOx (μmol/L)							
FO	32.17 (26.70, 37.64)	—	−0.76 (−8.08, 6.56)	—	−0.68 (−6.80, 5.45)	—	−0.57 (−6.25, 5.11)
HOS	31.99 (25.96, 37.24)	—	−2.43 (−7.33, 2.48)	—	1.13 (−4.56, 6.82)	—	−2.94 (−8.84, 2.97)
HLS	32.38 (25.84, 38.92)	—	−3.70 (−7.60, 0.20)	—	−1.80 (−5.00, 1.46)	—	−3.53 (−10.0, 2.97)
AO	33.43 (24.09, 42.76)	—	−5.30 (−13.56, 3.00)	—	−6.44 (−15.03, 2.14)	—	−4.56 (−11.33, 2.17)
Plasma nitrite (nmol/L)							
FO	234.00 (197.12, 270.90)	—	−1.19 (−32.44, 30.07)	—	−0.56 (−37.68, 36.56)	—	−9.46 (−31.38, 12.46)
HOS	237.04 (201.51, 272.54)	—	9.43 (−20.97, 39.82)	—	−4.75 (−29.27, 19.77)	—	−16.06 (−40.46, 8.34)
HLS	226.1 (180.42, 271.71)	—	−3.00 (−43.37, 37.37)	—	−17.33 (−45.24, 10.57)	—	−4.90 (−34.67, 24.88)
AO	225.2 (184.94, 265.42)	—	−7.84 (−31.56, 15.87)	—	3.20 (−29.60, 36.0)	—	−5.76 (−28.81, 17.29)
SBP (mm Hg)							
FO	120.4 (111.6, 129.2)	5.6 (1.8, 9.3)	6.8 (3.3, 10.3)	2.4 (−1.7, 6.5)	7.6 (3.6, 11.7)	3.1 (−1.5, 7.7)	5.0 (0.4, 9.6)
HOS	116.7 (108.2, 125.2)	7.4 (2.5, 12.3)	8.5 (3.8, 13.2)	5.9 (−0.4, 2.1)	8.4 (1.8, 15.0)	7.4 (3.6, 11.1)	7.9 (3.3, 12.5)
HLS	121.5 (112.5, 130.6)	3.1 (−1.7, 7.8)	2.6 (−2.2, 7.4)	2.6 (−2.1, 7.2)	5.2 (−0.6, 10.9)	4.1 (−2.0, 10.2)	3.4 (−1.8, 8.6)
AO	120.8 (110.6, 131.0)	5.5 (0.9, 10.2)	6.0 (2.0, 10.0)	2.6 (−1.2, 6.5)	6.1 (3.4, 8.8)	6.1 (1.9, 10.3)	5.1 (1.8, 8.5)
DBP (mm Hg)							
FO	75.0 (68.0, 81.9)	−0.7 (−4.2, 2.8)	−0.3 (−4.3, 3.7)	−2.0 (−5.6, 1.5)	0.6 (−4.0, 5.2)	1.0 (−3.1, 5.1)	0.2 (−3.6, 4.1)
HOS	70.6 (64.4, 76.9)	2.0 (−1.0, 5.2)	2.7 (−1.7, 7.1)	2.4 (−1.7, 6.5)	3.5 (−0.4, 7.4)	4.2 (0.6, 7.7)	4.3 (1.5, 7.1)
HLS	74.7 (68.3, 81.2)	1.0 (−2.3, 4.4)	−1.1 (−4.3, 2.1)	−0.2 (−4.0, 3.6)	1.4 (−2.1, 4.8)	1.5 (−2.0, 5.1)	1.6 (−1.6, 4.8)
AO	74.3 (66.6, 82.0)	0.6 (−2.9, 4.2)	0.2 (−2.8, 3.2)	−0.7 (−3.7, 2.2)	2.4 (0.1, 4.8)	1.1 (−1.7, 4.0)	2.1 (−0.0, 4.3)
DVP-SI (m/s)							
FO	8.5 (7.5, 9.55)	—	−1.0 (−1.6, −0.3)	—	−1.0 (−2.1, −0.0)	—	−1.3 (−2.2, −0.4)
HOS	8.2 (7.2, 9.2)	—	−1.3 (−2.2, −0.5)	—	−1.4 (−2.2, 0.5)	—	−1.0 (−1.7, 0.2)
HLS	9.1 (7.9, 10.3)	—	−1.9 (−3.0, −0.8)	—	−2.2 (−3.2, −1.1)	—	−1.8 (−3.2, −0.4)
AO	8.8 (7.7, 9.5)	—	−1.9 (−2.9, −1.0)	—	−1.6 (−2.6, −0.6)	—	−1.32 (−2.1, −0.6)
DVP-RI (%)							
FO	70.9 (65.8, 76.1)	—	−4.6 (−10.3, 1.1)	—	−2.9 (−11.3, 5.4)	—	−3.6 (−10.2, 3.1)
HOS	67.5 (62.4, 72.6)	—	−7.5 (−15.8, 0.9)	—	−2.1 (−9.5, 5.4)	—	−0.9 (−9.8, 8.1)
HLS	69.8 (63.4, 76.3)	—	−2.6 (−8.0, 2.8)	—	−1.8 (−8.1, 4.5)	—	−1.0 (−7.9, 5.9)
AO	71.4 (64.7, 78.0)	—	−10.6 (−18.8, −2.4)	—	−8.6 (−16.9, −0.3)	—	−3.7 (−10.5, 3.0)

1 All values are means; 95% CIs in parentheses. Analyzed by 2-factor repeated-measures ANOVA; *n* = 16. A dash indicates that no measurement was made at that time point. No differences at baseline were observed for each individual metabolite or vascular measure. With the exception of plasma nitrite (*P* > 0.05), all measurements changed over time (total NOx, DVP-RI, and DBP, *P* < 0.05; DVP-SI and SBP, *P* < 0.0001). There was neither a treatment effect nor a treatment × time interaction for any of the metabolites or vascular measures. AO, algal oil meal; DBP, diastolic blood pressure; DVP-RI, digital volume pulse–reflection index; DVP-SI, digital volume pulse–stiffness index; FO, fish-oil meal; HLS, high–linoleic acid sunflower oil meal; HOS, high–oleic acid sunflower oil meal; NOx, nitric oxide metabolite; SBP, systolic blood pressure.

### 8-Isoprostane F_2α_

**FIGURE 5.**
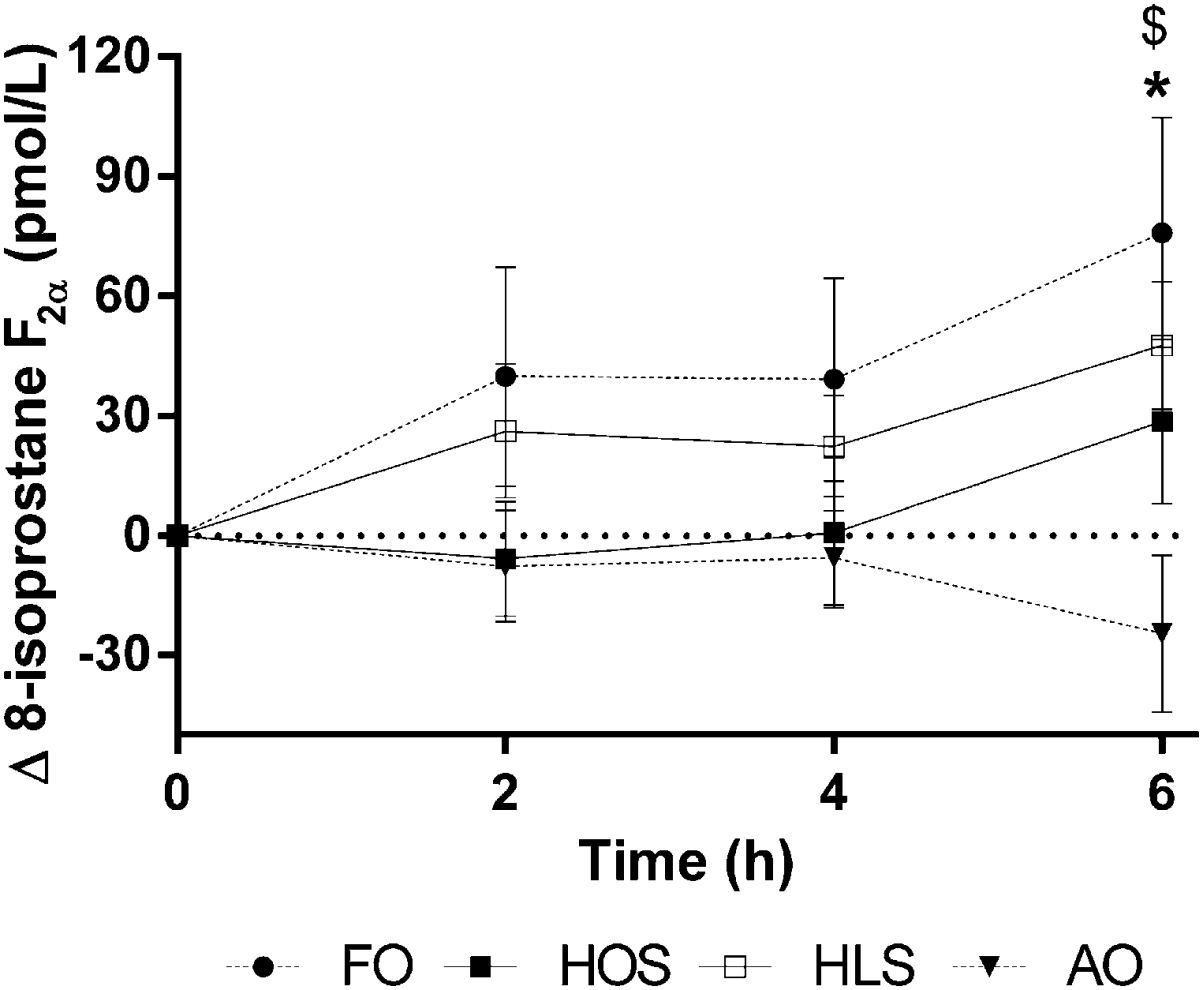
Change in plasma 8-isoprostane F_2α_ concentrations after 4 high-fat test meals. Values are mean (±SEM) changes relative to fasting baseline values at 2, 4, and 6 h after test meals; *n* = 16. Data were analyzed by 2-factor repeated-measures ANOVA with a Bonferroni post hoc test to determine which meals differed from control and at which time points. There were significant effects of time (*P* < 0.05) and a treatment × time interaction for values unadjusted for baseline (*P* < 0.05), and there was a trend for a treatment effect on change from baseline data (*P* = 0.052). Mean values after FO were increased relative to HOS and, after AO, were decreased relative to HOS. FO compared with HOS: *P < 0.05; AO compared with HOS: ^$^*P* < 0.05. AO, algal oil meal; FO, fish-oil meal; HLS, high–linoleic acid sunflower oil meal; HOS, high–oleic acid sunflower oil meal.

There was a statistically significant time effect for plasma 8-isoprostane F_2α_ concentrations (*P* < 0.05), reflecting postprandial increases after HOS, HLS, and FO, although no increase was observed after AO (**Figure 5**). A significant treatment × time interaction (*P* < 0.05) for data unadjusted for baseline values reflected reduced concentrations at 6 h after AO and increased concentrations following FO relative to HOS (post hoc statistics shown in the Figure 5 legend). No differences in postprandial 8-isoprostane F_2α_ concentrations were observed between HOS and HLS.

## DISCUSSION

This study investigated the effects of consuming high-fat meals of varying unsaturated fatty acid composition to test the hypothesis that meals enriched with LC n−3 PUFAs—either fish oil (EPA and DHA) or algal oil (DHA only)—result in more favorable vascular and oxidative stress responses postprandially compared with a control high-MUFA meal. In contrast, the postprandial response to a meal enriched with n−6 PUFAs was not expected to differ from the response to the control high-MUFA meal. High–oleic acid sunflower oil was used as the comparator because it has been shown to induce a pronounced lipemia and to impair flow-mediated dilatation (15, 17, 28), a gold-standard measure of endothelium-dependent vasodilation in the brachial artery. As expected, the fatty acid composition of the plasma was altered postprandially to reflect that of the meal composition (Figure 2). We investigated an older male population who had high-normal concentrations of fasting triacylglycerol and were therefore likely to have a more pronounced postprandial lipemia (29). Part of the broader aim of this project was complementary in vitro research using triglyceride-rich lipoprotein fractions isolated postprandially from study participants (results unpublished). Therefore, a greater fat load (75 g) to that previously used by our group (50 g) was administered to ensure a pronounced lipemic response and optimum harvesting of postprandial lipoproteins, and this was well tolerated by participants. Postprandial plasma triacylglycerol concentrations were marginally reduced after FO, but not AO, relative to the HOS reference meal. We have previously reported in younger subjects that purified EPA-rich FO did not reduce postprandial triacylglycerol 6 h after a high-fat meal (24, 30). Although it is well established that chronic supplementation with FO reduces postprandial lipemia (31–33), most studies have not shown any reduction in relative postprandial triacylglycerol response after a single FO test meal (11, 14, 30, 34–36) apart from those that used larger doses of EPA + DHA (37, 38).

Apart from its effects on plasma triacylglycerol concentrations, FO had no other effects on plasma lipids postprandially compared with the control HOS meal. However, AO clearly influenced circulating NEFA concentrations. Postprandially, stored triacylglycerol lipolysis is suppressed because of the inhibitory action of insulin on hormone-sensitive lipase so that plasma NEFA concentrations are reduced (39, 40). Here, the characteristic decrease in plasma NEFAs was not as marked after the AO meal relative to the HOS reference meal. Although purely speculative at this stage, potential mechanisms may involve an increase in spillover fatty acids escaping from lipoprotein lipase action on chylomicron triacylglycerol or reduced insulin secretion/action, resulting in diminished suppression of adipocyte triacylglycerol lipolysis. The role of insulin in the diminished suppression of circulating NEFA concentrations after AO was not explored in the current study because more frequent blood sampling time points would be necessary to accurately measure plasma insulin concentrations during the early postprandial period. In contrast, the NEFA response to FO was similar to that seen with HOS. A greater reduction in NEFAs after an FO compared with a control meal has been reported previously, but a lower EPA-to-DHA ratio and lower total fat content were used in the test meal compared with the current study (41). To summarize the findings regarding postprandial lipids, subtle differences in triacylglycerol response to FO and AO are likely to be of little clinical importance regarding the therapeutic use of prescribed n–3 fatty acid products, and there was a clear inhibition of the early postprandial suppression of circulating NEFA concentrations after AO relative to HOS control. Mechanisms for this latter effect are unclear.

Other studies have demonstrated that EPA alone (24) and in combination with DHA (as fish oil) (11, 42, 43) enhances vasodilation and vascular function in the postprandial state. In this study, there was no effect of either LC n−3 PUFA meal on AIx_ao_, AIx_br,_ DVP-SI, or DVP-RI relative to the control, which contrasts with Chong et al (41), who reported a marginally enhanced postprandial reduction in AIx and DVP-SI after FO. HLS inhibited the reduction in central AIx observed postprandially relative to HOS, FO, and AO. However, this result should be interpreted with caution because it is well known that aortic pulse wave reflection may vary with heart rate independently of changes in arterial tone. Specifically, a reduction in heart rate tends to be associated with an increase in central AIx resulting from the lengthening of systole (44). Therefore, the increase in AIx_ao_ observed here may reflect the trend for a greater reduction in heart rate after HLS (data not shown, *P* = 0.09) compared with control, rather than an increase in arterial stiffness.

Plasma NOx concentrations were reduced after all 4 meals, with no differences between treatments, and plasma nitrite did not change after the high-fat meals. In contrast, an improvement in acetylcholine-induced vasodilation after FO (5.4 g EPA + DHA) consumption accompanied by increased postprandial nitrite concentrations has been reported in a study in healthy men (11). We used a gas phase chemiluminescence reaction between nitric oxide and ozone to quantify nitrite and total NOx, which is thought to be more sensitive than the commercially available kits.

Oxidative stress contributes to the development of CVD (45), and high concentrations of urinary or plasma F2-isoprostanes, resulting from nonenzymatic oxidation of arachidonic acid, are suggested to be nonspecific indicators of CVD (46). Previous studies demonstrated increased plasma 8-isoprostane F_2α_ concentrations after high-fat meals containing oleic acid (15), with further increases evident when EPA was incorporated (24). Here, FO increased 6-h plasma 8-isoprostane F_2α_ relative to the HOS reference meal. Intriguingly, AO reduced 8-isoprostane F_2α_ concentrations compared with HOS and FO. This novel finding suggests that EPA and DHA may have opposing effects on free radical–induced lipid peroxidation associated with postprandial lipemia. However, this explanation may be misleading because the increase in plasma 8-isoprostane F_2α_ after FO is not consistent with the reduced postprandial triacylglycerol response observed after FO relative to AO, which would be expected to result in lower levels of oxidative stress. Results presented here and previously (24) suggest that fish oil (more specifically EPA) may induce transient increases in nonenzymatic oxidation of arachidonic acid when consumed with a high-fat meal. Competition between EPA and arachidonic acid for oxidative enzymes, such as cyclooxygenases 1 and 2, lipoxygenases, and cytochrome P450 mono-oxygenases, may lead to a greater availability of arachidonic acid for nonenzymic oxidation, leading to increased 8-isoprostane F_2α_ production (47). In the case of the AO meal, the reduced isoprostane concentrations might be explained by replacement of arachidonic acid by DHA in the lipid membrane (48), potentially leading to reduced substrate availability for 8-isoprostane F_2α_ generation. Further research, including measurement of EPA- and DHA-derived isoprostanes, should be carried out to clarify the physiologic significance of these observations.

Strengths of this research include the design (randomized, controlled, double-blind crossover trial) and the use of sensitive, specific techniques to measure study outcomes (eg, plasma 8-isoprostane F_2α_ and NOx). The target population was healthy but displaying signs of a metabolically “risky” phenotype and therefore more likely to be discriminatory in terms of the differential effects of unsaturated fatty acids. The study is limited by inclusion of men only, and thus the effects of meal fatty acid composition cannot be extrapolated to women. Clearer data on the relative effects of EPA and DHA could be obtained if a similar study were carried out with pure sources of EPA compared with DHA, but a pure source of EPA triacylglycerol was not available when the study commenced. Because of technical difficulties, we were unable to obtain Arteriograph*24* vascular data from 2 of the 16 completing participants, and therefore the study may have been statistically underpowered for augmentation index and blood pressure measures.

In summary, the results of this study indicate that high-fat meals of varying unsaturated fatty acid composition differentially influence the postprandial metabolic and vascular response. Although EPA and DHA belong to the same fatty acid class and have similar structures, it has been reported that they exert different and often opposite effects on vascular function both in vitro and in vivo (16). The main novel finding is that postprandial 8-isoprostane F_2α_ production is lower after consumption of a meal enriched with DHA (of algal origin), whereas an FO meal (EPA + DHA) has the opposite effect. This implies that EPA and DHA generate metabolites that exert divergent effects on oxidative pathways in the vasculature (49) and opens up an important line of investigation into potential mechanisms mediating the vasculo-protective properties of LC n−3 PUFAs during the postprandial state. In conclusion, although research into the clinical significance of the dietary EPA-to-DHA ratio is in its preliminary stages, the current findings suggest that consumption of DHA-only sources may have distinct effects on lipid metabolism compared with EPA + DHA sources with important implications for cardiovascular health.

## Supplementary Material

Supplemental data

## Footnotes

5 Abbreviations used: AIx, augmentation index; AIx_ao_, central augmentation index; AIx_br_, peripheral augmentation index; AO, algal oil meal; CVD, cardiovascular disease; DVP, digital volume pulse; DVP-RI, digital volume pulse–reflection index; DVP-SI, digital volume pulse–stiffness index; FO, fish oil; HLS, high–linoleic acid sunflower oil; HOS, high–oleic acid sunflower oil; LC, long chain; NEFA, nonesterified fatty acid; NOx, nitric oxide metabolites.
