# Supplementary material for: High-fat meals rich in EPA plus DHA compared with DHA only have differential effects on postprandial lipemia and plasma 8-isoprostane F2α concentrations relative to a control high–oleic acid meal: a randomized controlled trial1
Source: Am J Clin Nutr. 2014 Aug 6;100(4):1019–28. doi: 10.3945/ajcn.114.091223 (PMC4163792; doi:10.3945/ajcn.114.091223)
Supplement: Supplemental data [file supp_100_4_1019__index.html]

High-fat meals rich in EPA plus DHA compared with DHA only have differential effects on postprandial lipemia and plasma 8-isoprostane F2α concentrations relative to a control high–oleic acid meal: a randomized controlled trial — High-fat meals rich in EPA plus DHA compared with DHA only have differential effects on postprandial lipemia and plasma 8-isoprostane F2α concentrations relative to a control high–oleic acid meal: a randomized controlled trial — Supplemental data 

# High-fat meals rich in EPA plus DHA compared with DHA only have differential effects on postprandial lipemia and plasma 8-isoprostane F2α concentrations relative to a control high–oleic acid meal: a randomized controlled trial

## Supplemental data

**Files in this Data Supplement:**

- Supplemental data - Table 1
