# Supplementary material for: High-fat meals rich in EPA plus DHA compared with DHA only have differential effects on postprandial lipemia and plasma 8-isoprostane F2α concentrations relative to a control high–oleic acid meal: a randomized controlled trial1
Source: Am J Clin Nutr. 2014 Aug 6;100(4):1019–28. doi: 10.3945/ajcn.114.091223 (PMC4163792; doi:10.3945/ajcn.114.091223)
Supplement: Supplemental data [file 114.091223_ajcn091223SupplementaryData1.doc]

**Supplemental Table 1**

**Baseline plasma TAG and NEFA concentrations**

|  | **Plasma NEFA (mmol/L)^1^** | **Plasma TAG (mmol/L)^2^** |
| --- | --- | --- |
| *FO* | 0.64 (0.57, 0.70) | 1.66 (1.34, 2.04) |
| *HOS* | 0.61 (0.54, 0.68) | 1.54 (1.29, 1.82) |
| *HLS* | 0.60 (0.54, 0.67) | 1.46 (1.25, 1.71) |
| *AO* | 0.53 (0.45, 0.60) | 1.81 (1.50, 2.19) |

^1^Values are means with 95% CI. ^2^ Values are geometric means with 95% CI. Analysed by repeated measures ANOVA; n = 16. No differences at baseline were observed for plasma TAG or NEFA between the groups. AO: algal oil meal; FO: fish oil meal; HLS: high linoleic acid sunflower oil meal; HOS: high oleic sunflower oil meal; NEFA: non-esterified fatty acids; TAG: triacylglycerol.
